# e-Diagnosis in Medical Parasitology

**DOI:** 10.3390/tropicalmed5010008

**Published:** 2020-01-03

**Authors:** Harsha Sheorey

**Affiliations:** Medical Microbiologist, St Vincent’s Hospital, Melbourne 3065, Australia; harsha.sheorey@svha.org.au

**Keywords:** e-Diagnosis, morphologist, molecular parasitology

## Abstract

Over the past decade or two, the teaching of laboratory diagnostic parasitology has been neglected in Australasia, as parasitic infections are relatively uncommon. As a consequence, expertise in medical parasitology is dwindling. A team of international experts (including Professor John Goldsmid) has been formed to help in the diagnosis of human parasitic infections. The team includes experts from Australia, Europe, South Africa and the USA. Some senior members of the team are excellent morphologists, and we have both human and veterinary parasitologists who help with molecular diagnosis in difficult cases.

Professor John Goldsmid has been a doyen of medical parasitology in Australia for many years. I was honored to meet John during our inaugural parasitology master class organized by the Australian Society for Microbiology (ASM) in Hobart in 2009 (Figure 1). Goldsmid’s knowledge, dedication and teaching in this field must be carried on for the future generations of health professionals. His attitude towards learning is exemplary (see response to Case 4 below).

Over the past decade or two, the teaching of laboratory diagnostic parasitology has been neglected in Australasia because parasitic infections are relatively uncommon. As a consequence, the expertise in medical parasitology is dwindling. While on a sabbatical at the Centre for Disease Control and Prevention in Atlanta (CDC), USA, I came across a method of diagnosing parasites that the parasitology department there had adopted to help less experienced health professionals. A picture of an unknown malarial parasite from Africa was sent to the parasitologists on a smart phone, and, after looking at the image, they were able to send back a diagnosis of malarial parasite to a species level. This method could easily be adopted in Australia, I thought. Thus, a team of experts who have helped in diagnosing and managing parasitic infections was put together.

## 1. Why e-Diagnosis?

- Less importance/attention is given to parasitic diseases in humans in the developed world (including Australia).
- The re-distribution of parasites due to human/animal movement and environmental and practice changes has brought parasites into the developed world.
- There are declining numbers of experts in this field, especially morphologists.
- The internet ‘savvy’ public who ‘self-diagnose’ want to ‘confirm’ their thoughts.

## 2. Who Is the Team?

**Figure 2:**
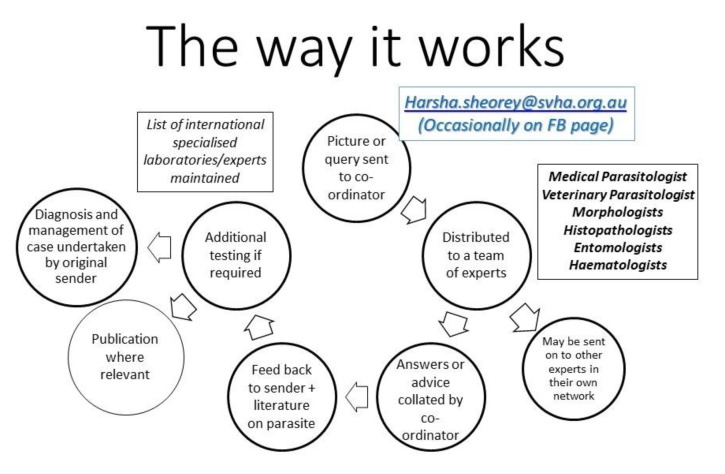
The way e-Diagnosis works.

The team includes parasitologists and other experts from Australia, Europe, South Africa, and the USA. Some senior members of the team are excellent morphologists, and we have both human and veterinary parasitologists who help with molecular diagnosis (Figure 2). The author acts as a coordinator of the group. The team has helped diagnose a few unusual parasites, leading to publications [1,2,3,4].

## 3. What Kind of Material/Advice is Sought?

Material:

- Images for identification (macroscopic and microscopic)
- Histology sections of tissues with possible parasites

Advice:

- Molecular identification/availability
- Interpretation/availability of serology for parasites
- Advice on testing to exclude parasites
- Management of parasitic infections (treatment and follow-up)—OzBug (Australian email group of interested clinical experts)
- Advice on/to “internet-savvy” patient concerns

## 4. Type of Parasites Received from Intestine, Tissues or Blood

- Protozoa
- Nematodes
- Trematodes
- Cestodes
- Arthropods
- Pseudo-parasites and artefacts

## 5. Cases

### 5.1. Case 1: Lump in the Back (Melbourne, VIC, Australia)

**Figure 3:**
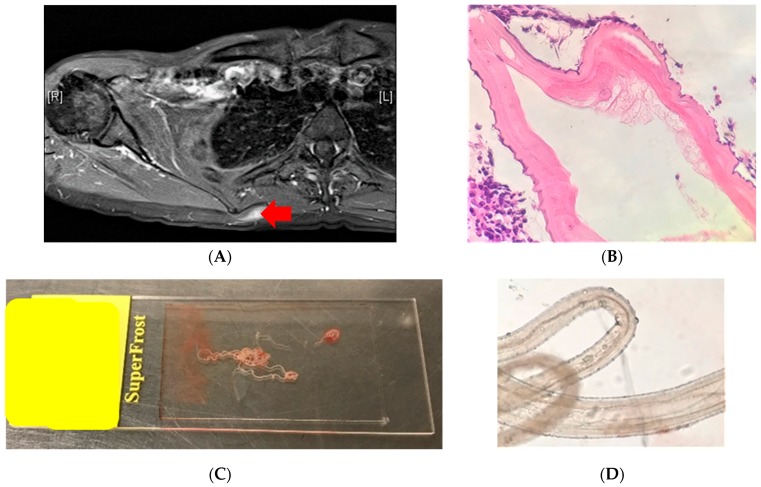
(**A**) PET/CT scan of back showing a lump in the back (red arrow). (**B**) Histology section of the biopsy showing a nematode helminth with features of a filarial worm. (**C**) Intertwined worm(s) fell out of the surgically removed lump. (**D**) On exam, these were found to be two, a thicker female and a thinner male.

A lump in the back (right scapular region) (Figure 3A) in a young women was biopsied to rule out sarcoma. Its histology showed a helminth with morphological features suggestive of a filarial worm—*Onchocerca* or *Dirofilaria* species (Figure 3B). This prompted the aid of an infectious diseases consult, and history-taking revealed that the patient was a veterinarian with extensive exposure to various animals both in Australia and Africa. Apart from swelling, the patient felt ‘movement’ inside their nodule. A lumpectomy was performed, and, in the laboratory, the worm fell out of the lump (Figure 3C). On examination, this was confirmed to be a filarial worm with features of *Onchocerca* species (cuticular rings and subcuticular striae) (Figure 3D), most likely a zoonotic *Onchocerca lupi* or *Onchocerca volvulus*.

e-Diagnosis members views:

Initial histology: Experts were convinced the section had features of a filarial helminth (ridges and internal structures), most likely a zoonotic *Onchocerca* species or *Dirofilaria* species.

After the worm was extracted, experts were convinced that the morphology was most consistent with *Onchocerca* species. A PCR-based sequencing method confirmed it to be *O. volvulus* (1).

### 5.2. Case 2: Lump in the Axilla (Brisbane, QLD, Australia)

**Figure 4:**
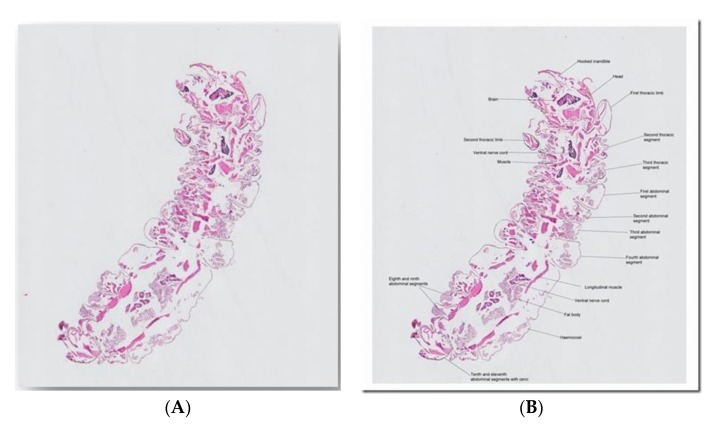
(**A**) Picture of histological section of ‘worm’ sent to the e-Diagnosis team. (**B**) The same picture that was labelled and sent back to the team by Professor Anderson, showing various morphological features that are consistent with an endopterygota larva.

A 23 year old from Brisbane, Australia, presented with an axillary mass for six months. He noticed movement within, and a small worm was expressed. This was placed in formalin. The dimensions were 1.2 mm wide by 3.85 mm long (Figure 4). No relevant travel history was noted.

e-Diagnosis member view:

*Professor Emeritus Don Anderson (previously Challis Professor of Zoology at Sydney University)*

‘An endopterygota larva, most probably a beetle larva with hooked mandibles. A lot of beetle larvae burrow into organic substrates. It’s a pity histopathologists almost invariably section things that are more easily identified intact.’

### 5.3. Case 3: Larvae, Adults and Eggs in CSF/Brain (Adelaide, SA, Australia)

**Figure 5:**
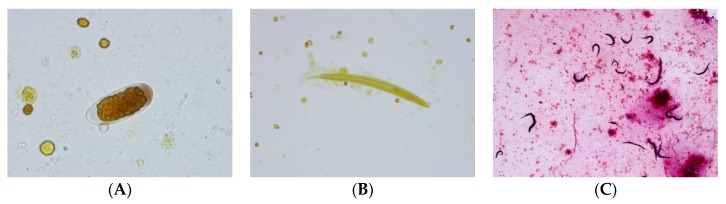
(**A**) Egg of parasite in CSF resembling the *Trichostrongylus* species (~50 × 20 µm). (**B**) larva of parasite in CSF resembling *Strongyloides* species (~250 µm). (**C**) Post-mortem brain tissue showing adult worms, larvae and eggs of the helminth (details and additional pictures in Reference 2).

A 73 year old woman was admitted to a regional South Australia hospital (past history with unspecified abdominal pain). The patient was on low dose methotrexate and etanercept for Rheumatoid arthritis (RA). She was transferred to Royal Adelaide Hospital with deteriorating neurological signs. The Cerebro-spinal fluid (CSF) taken showed 280 polymorphs, 18 monocytes, no bacteria seen, high protein, and virology and bacterial PCR and *Cryptococcal* antigen all negative. The CSF taken two days later showed 2500 polymorphs, 34 monocytes, no bacteria seen; Diff-Quik (modified Romanowsky Stain) showed “few eosinophils,” and no trophozoites. *Acanthamoeba* cultures were set up. Patient died less than a week after admission, and a post mortem examination was conducted. The CSF at post mortem showed 1700 polymorphs (PMN) (few eosinophils), a very high protein level, and numerous helminth larvae and eggs (Figure 5A,B). *Strongyloides* and *Angiostrongylus* serology was negative. A brain biopsy demonstrated the presence of a helminth. PCR-based sequencing confirmed it to be *Halicephalobus gingivalis* (Figure 5C).

e-Diagnosis members views:

What follows is a summary of the email discussion that occurred after images were sent to the team. The experts’ names are in brackets.

- The structure of the esophagus and ratio to gut may also help us. The two illustrated larvae could just be rhabditiform and hence no notching of tail; there does seem to be a darker region near the genital primordium zone in one photo, but we also lack ability to gain more definition of these key points. (Norbert Ryan).
- The egg looks very much like a *Trichostrongylus* egg (but the size of the eggs is too small), and the larvae have features that are reminiscent of that species also, particularly the wavy pattern of the intestine (John Walker).
- First of all, very few documented cases (of *Strongyloides*) in the brain/CSF. Have no explanation for eggs/rhabditiform larvae in this site (Lynne Garcia).
- I don’t think it is the usual suspects, namely *Strongyloides* or *Angiostyrongylus*, on morphological grounds. Eggs are not normally seen in angiostrongyliasis. They look most like stages of *Trichostrongylus*, but that infection does not disseminate, or at least there are no reports thereof. Thus, I think this is a very unusual aberrant infection with *Trichostrongylus* or similar nematode that is not normally found in humans (John Frean).
- I found a lovely description of all stages of *Halicephalobus gingivalis* in Anderson et al. (1998). This is now regarded as the correct name for the parasite. The drawings are very consistent with what is in the images. Note the elongated eggs, the recurved ovaries, and of course the rhabditoid esophagus (Rick Speare).
- Please don’t waste the nematodes you have by rushing in and using them immediately for PCR. They should be morphologically described first; then do PCR. Additionally, some should be kept in case it is a new species. Don’t forget the light microscopy and measurements, as well as SEM. Of course, the latter looks more spectacular but usually carries less taxonomic weight (Rick Speare).
- The sequence results have come back, and the worms are positive for *Halicephalobus gingivalis*. The 28s D2/D3 primers from Nadler’s 2003 paper were used and got the sequence found below (2). This sample was 99% identical to GenBank accession AY294177 over 786 base pairs (Anson Kohler).
- The combination of case details, plus description of the nematodes, plus PCR gives the maximum value. Fabulous effort! Good example of the value of a collaborative dispersed group in diagnosing unusual parasites! (Rick Speare)

### 5.4. Case 4: Worm Passed in Feces (Wollongong, NSW, Australia)

**Figure 6:**
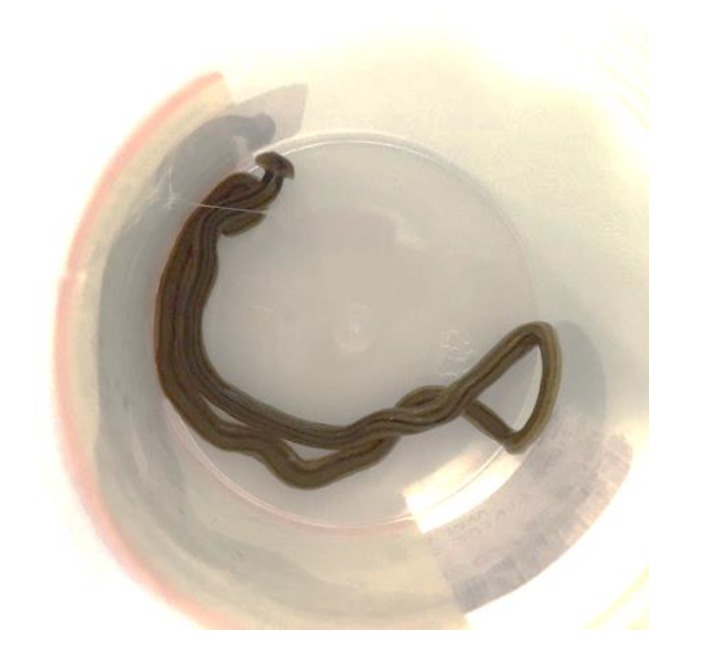
Worm thought to have wriggled out of feces on the floor in an incontinent patient.

*From Dr. Peter Newton, Pathologist in Wollongong*: This worm was sent to us for identification from one of our regional hospitals 80 km away. The history that we received from the ID physician who sent it to us was that of a 75 year old female patient admitted with severe Clostridium difficile-associated disease (CDAD). Her condition has apparently been slowly improving. Yesterday, she was incontinent of feces on the ward floor, and this worm was seen wriggling out of her feces on the floor (Figure 6).

Based on a Google search, we think that it is a “hammerhead worm” of the *Bipalium* genus. We assume it was on the floor and crawled into her feces, or the feces landed on the worm on the floor. It was a rather wet morning on the New South Wales (NSW) coast yesterday, so presumably came onto the ward on someone’s shoe. Would value your opinion.

e-Diagnosis members view/comments:

*Dr. John Walker (ex-Head of Parasitology, Westmead Hospital, Sydney)*

The worm is *Bipalium kewense*, first described at Kew Gardens but originally from Southeast Asia. Now has a cosmopolitan distribution as a consequence of trade in botanical specimens. It’s a predatory, free-living planarian.

*Prof John Goldsmid (UTas, Hobart)*

My goodness—we live and learn, even in retirement!! I have never seen this worm or heard of a “hammerhead worm.” Peter’s tentative identification seems reasonable and probable. I congratulate him on his detective work.

## 6. Limitations of This Diagnostic Procedure

- Poor images may be sent:
  - Not enough images: multiple images at different magnification levels and that cover key parts of the parasite are not always sent.
  - Poor quality photos that are out of focus or focused on wrong part may be sent. The new smart phones take very good images, so this should not be an excuse.
- No relevant history: Age, epidemiology, travel, exotic food, contact with animals, medication, immune status are all important, and, quite often, this is not communicated.
- Arthropods or large worms received in formalin and sectioned: Arthropods and large worms are better photographed whole and not sectioned. When stored in 70% alcohol, formalin makes specimens ‘brittle.’
- ‘Degenerate’ or dying parasite (histology-section): Morphology may not be typical enough to make a diagnosis.
- Not enough follow up material: The specimen or multiple images should be held onto if required for further identification.
- Direct contact with non-scientific public (via social media and internet):
  - Abuse: Some members of the public do not like answers that they do not want to hear and can get abusive.
  - Legal aspects: Advice/opinions given over limited information and presented this way should be taken in the context of clinical pictures. It should be made clear that this is an opinion, and a clinical judgment should be made by the treating doctor. Advice on treatment (drugs) should be avoided over internet/social media, as other medical history information needs to be taken in consideration. Wrong interpretation/understanding can lead to legal ramifications.

## 7. Summary and the Future

Given the decreasing expertise in medical parasitology, this type of diagnostic help will become common and convenient. A team of experts can be put together from various parts of the world, and images and questions can be shared over electronic media. Additionally, sending bits of specimens by post to experts anywhere in the world is now possible in case molecular techniques are required for diagnosis. However, the legal aspects of giving advice and possible abuse over electronic or social media should be kept in mind.

## Figures and Tables

**Figure 1:**
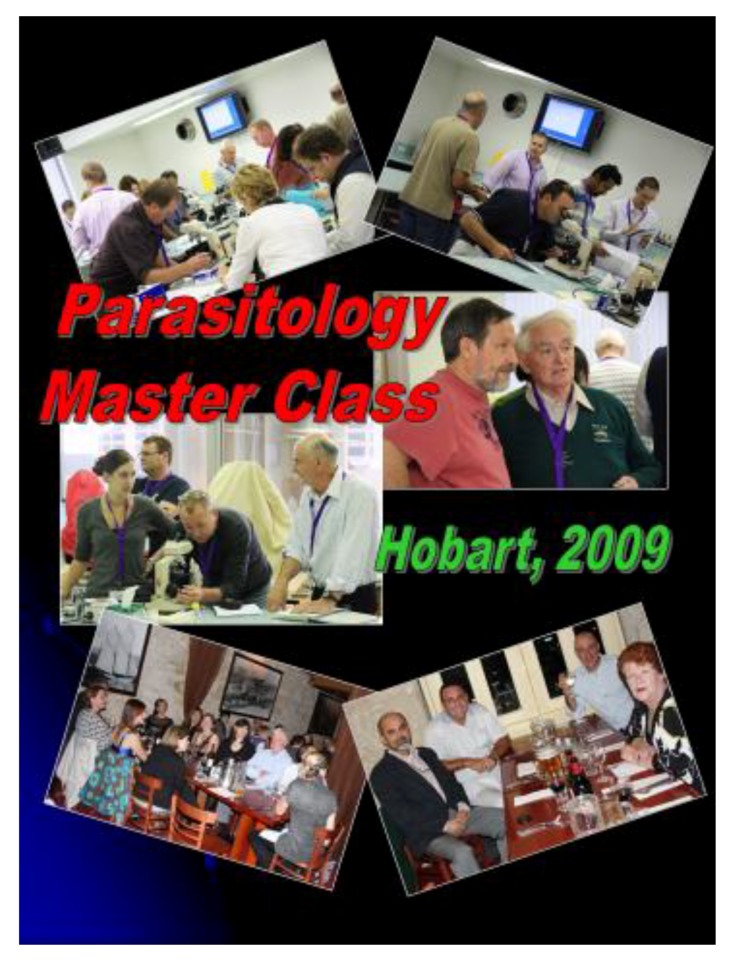
The inaugural Australian parasitology master class.
